# The knowledge and attitudes of breast self-examination and mammography in a group of women in a rural area in western Turkey

**DOI:** 10.1186/1471-2407-6-43

**Published:** 2006-02-24

**Authors:** Pınar Erbay Dündar, Dilek Özmen, Beyhan Öztürk, Gökçe Haspolat, Filiz Akyıldız, Sümeyra Çoban, Gamze Çakıroğlu

**Affiliations:** 1 Public Health Department, Faculty of Medicine, Celal Bayar University, Manisa, Turkey; 2 Dep. of Public Health Nursing, School of Health, Celal Bayar University, Manisa, Turkey

## Abstract

**Background:**

Breast cancer appears to be a disease of both the developing and developed worlds. Among Turkish women, breast cancer is the second leading cause of cancer-related deaths. The aims of this cross-sectional study were to determine levels of knowledge about breast cancer and to evaluate health beliefs concerning the model that promotes breast self- examination (BSE) and mammography in a group of women aged 20–64 in a rural area of western Turkey.

**Methods:**

244 women were recruited by means of cluster sampling in this study. The questionnaire consisted of sociodemographic variables, a risk factors and signs of breast cancer form and the adapted version of Champion's Health Belief Model Scale (CHBMS). Bivariate correlation analysis, Chi square test, Mann-Whitney U test and logistic regression analysis were performed throughout the data analysis.

**Results:**

The mean age of the women was 37.7 ± 13.7. 49.2% of women were primary school graduates, 67.6% were married. Although 76.6% of the women in this study reported that they had heard or read about breast cancer, our study revealed that only 56.1% of them had sufficient knowledge of breast cancer, half of whom had acquired the information from health professionals.

Level of breast cancer knowledge was the only variable significantly associated with the BSE and mammography practice (p = 0.011, p = 0.007). BSE performers among the study group were more likely to be women who exhibited higher confidence and perceived greater benefits from BSE practice, and those who perceived fewer barriers to BSE performance and possessed knowledge of breast cancer.

**Conclusion:**

By using the CHBMS constructs for assessment, primary health care providers can more easily understand the beliefs that influence women's BSE and mammography practice.

## Background

Breast cancer appears to be a disease of both the developing and developed worlds. It is the leading type of cancer in women. Among Turkish women, breast cancer represents 24.1% of all cancers seen in women and is the second leading cause of cancer-related deaths. About 2390 new cases of breast cancer were diagnosed in 1999 in Turkey [1]. The risk of breast cancer increases with age. The primary factors that increase risk of breast cancer in women include certain inherited genetic mutations, a personal or family history of breast cancer, and biopsy-confirmed hyperplasia [2]. Other factors that increase breast cancer risks include a long menstrual history (menstrual periods that started early and/or ended late in life), obesity after menopause, recent use of oral contraceptives, postmenopausal hormone therapy, never having had children or having the first child after age 30, ethnicity characteristics, exposure to radiation, or consumption of one or more alcoholic beverages per day [2,3]. High breast tissue density (which is a mammographic measure of the amount of glandular breast tissue relative to fatty tissue in the breast)is a known feature that reduces the breast cancer detection rates in clinical breast examination (CBE) and mammogram. Factors that decrease breast cancer risks include breastfeeding, moderate or vigorous physical activity, and the maintenance of a healthy body weight [2].

Early detection and prompt treatment offer the greatest chance of long-term survival [4]. Mammography, clinical breast examination and breast self-examination (BSE) are the secondary preventive methods used for screening in the early detection of breast cancer [5]. Cancer screening tests play a pivotal role in reducing breast cancer related mortalities [6]. The American Cancer Society (ACS) recommends CBE and mammography in the early detection of breast cancer [7]. According to ACS recommendations, women should know how their breasts normally feel and report any breast changes promptly to their health care providers. BSE is an option for women starting from the early 20 s [2,8]. ACS no longer recommends BSE as there is reliable data that breast cancer detection through BSE does not increase survival rates. But, BSE seems to be an important viable optional substitute available in rural areas, where access to CBE and mammograms is difficult and might still detect breast cancer early enough for treatment which can be offered to prolong women's lives and reduce suffering. For younger women, BSE training and adherence is a gateway health promotion behaviour provides women with the knowledge that sets the stage for adherence to CBE and mammography screening guidelines later in life. Screening is linked to perceptions of risk, benefit, and barriers through a reasoning process that includes personal and social influences and attitudes [9]. However, only a few Turkish women do it. According to studies, only 27–39% of Turkish women had ever performed BSE, but Secginli reported that women performing BSE once a month constituted 5.5% of the population in Istanbul [10].

Women in their 20 s and 30 s should have a CBE as part of a periodic (regular) health examination by health professionals preferably every 3 years. After the age of 40, women should have a CBE every year, as recommended by the ACS [2]. Zincir reported that the percentage of women over the age of 40 having a professional breast examination every year was 21.1% the eastern region of Turkey [11].

Annual mammography is considered the most valuable tool for detecting breast cancer in the earliest possible stages, before the cancer has metastasized and when interventions are most effective and least invasive and debilitating. The decline in breast cancer mortality has been largely attributed to regular mammography screening practice [12]. The ACS recommends that women aged 40 and over should have a screening mammogram every year and should continue to do so for as long as they are in good health [2]. Mammography screening, can lower the mortality risk but it is still under-used among minorities [13]. The rate of undergoing a recommended mammography practice was 12.6 % in Secginli's study performed in Istanbul [14].

### Health belief model

The Health Belief Model (HBM) was one of the first models to adapt theories from the behavioural sciences in order to examine health related problems. It is still one of the most widely recognised and used models in health behaviour applications. This model was originally introduced by a group of psychologists in the 1950's to help explain why people would or would not use available preventive services, such as chest x-rays for tuberculosis screening and immunisations for influenza [15].

Many investigators studying beliefs related to cancer screening practices have used the HBM as a theoretical framework to study breast cancer screening behaviour such as BSE or mammography screening [12,16].

The HBM has frequently been applied to breast cancer screening [17]. The model stipulates that health-related behaviour is influenced by a person's perception of the threat posed by a health problem and by the value associated with his or her action to reduce that threat [18].

According to the HBM, a woman who perceives that she is susceptible to breast cancer and that breast cancer is a serious disease would be more likely to perform regular breast examinations. Similarly, a woman who perceives more benefits from and fewer barriers to BSE would be more likely to practice BSE. A woman who has an internal cue (body perception) or who has been exposed to an external cue (e.g., the positive influence of a health care provider or the media) would also more readily adopt BSE, as would a woman who wants to improve her health and who is confident of positive results [18,19].

The HBM consists of 6 concepts: (1) perceived susceptibility to an illness, (2) perceived seriousness of the illness, (3) perceived benefits for the presumed action, (4) perceived barriers for the presumed action, (5) confidence in one's ability, and (6) health motivation. Behaviour is also a result of the belief that a certain action will benefit the individual and that this benefit will outweigh any barriers [20].

The investigation of attitudinal components of health-related behaviour has been important. If attitudes related to health behaviour can be identified, health protection interventions for attitudinal change can be developed, and an increase in desirable health behaviour would result [21].

Despite being one of the few cancers which is able to be detected in its preclinical stage, BSE and mammography are still only practised by a low proportion of the population in our country, and this anomaly forms the basis of this study. The aims of this study were to determine knowledge levels about breast cancer, and to evaluate the health belief model about BSE and mammography in a group of 20–64 year-old women in Muradiye-Manisa, a rural area of western Turkey.

## Methods

### Sample

In this cross-sectional study, the data was collected from the rural area of Manisa, a city located in the western region of Turkey. The total study population consisted of 1,829 women aged between 20–64 years living in the Muradiye Health District in Manisa. The aim of the study was to reach at least 236 women with a 99.9% confidence level and 0.50 prevalence by using the Epi info 2000 statistical software. Cluster sampling method was performed proportional to the percentage of the three neighbourhood populations in Muradiye District. A total of 24 clusters were selected. It was decided that each cluster would consist of 10 houses and the first house of the cluster was selected randomly. After the first initially selected house, every subsequent forth house was selected (i.e 1st, 5th, 9th etc). For the women who were absent, the visits were repeated once. And if they weren't found on these second visits either, the neighbouring house was included in the study. As a result, 244 women were interviewed. When the visits were made to the selected houses, it was found that, in some houses, more than one female occupant aged 20–64 (daughters, mother-in-laws, aunts etc.) resided at the given address. The response rate of the study surpassed 100%, since all of the women aged 20–64 living in the selected houses were incorporated in the study. Although the advancement in age is a risk factor in breast cancer, women over 65 were not included in this study, due to a perceived risk of inadequate perception. Four trained intern doctors from the medical faculty collected the data in face-to-face interviews between January and February 2005. They made clear the confidentiality, benefits, risks, and future implications of the research. Data was then collected from those who verbally consented to participate. The study was ethically approved by the Manisa Province Health Directorate.

### Measurement

The questionnaire comprised of sociodemographic variables, a form regarding risk factors and signs of breast cancer and the measurement of the health belief model of breast cancer [see Additional file 1]. Sociodemographic measures, including characteristics such as the respondent's age, current marital status, level of education, income level, family type, health insurance, and migration state were assessed. The perceived income level was measured to identify the income level of the family since it is a simple marker for the determination of the economic level, and it was coded as sufficient = 1 or insufficient = 2.

The subjects were also asked if they had knowledge of breast cancer and if there were family members and/or friends with breast cancer histories. 24 questions were comprised to determine the individuals' level of knowledge of breast cancer; 15 of which were about risk factors and 9 about the signs and symptoms of breast cancer. The answers were 'true', 'false' and 'don't know'. The knowledge score was computed by totalling the number of correct answers for all 24 questions. The knowledge score was re-coded into dichotomous variables by taking the mean value as the cut off value to evaluate knowledge levels, coded sufficient = 1 and insufficient = 2. Univariete risk analysis was performed with the demographic, socioeconomic variables and knowledge levels. Logistic regression analysis was used to evaluate the significant variables with 95% CI (Confidence Interval) found in univariete analysis.

CHBSM was also applied to the subjects. The Health Belief Model Scale was developed in 1984 and was revised in later works by Champion [19,20,22]. It was validated for Turks by Secginli et al [10]. CHBSM is a 53-item, self-report measure, representing 8 scales, susceptibility (5 items); seriousness (7 items); benefits-BSE (6 items); barriers-BSE (6 items); confidence (11 items); health motivation (7 items); benefits- mammography (6 items); and barriers- mammography (5 items). All the items have 5 response choices ranging from strong disagreement (1 point) to strong agreement (5 points). All scales are positively related to screening behaviour, except for barriers which are negatively associated. Minimum and maximum values for sub-scales are susceptibility: (5–25), seriousness: (7–35), benefits-BSE: (6–30), barriers-BSE: (6–30), confidence: (11–55), health motivation: (7–35), benefits- mammography: (6–30), and barriers- mammography: (5–30).

### Statistical analysis

Bivariate correlation analysis, Chi square test, Mann-Whitney U test and logistic regression analysis were performed in the data analysis, by using SPSS v10.0 statistical package.

### Description of the sample

The women's mean age was 37.7 ± 13.7. 49.2% of women were primary school graduates, 67.6% were currently married, 61.1% had a sufficient income level and 91.0% were housewives (Table 1). In general women of the western region of Turkey are less traditional and better educated than those in the general population of Turkey (37.6% are primary school graduates and 33.6% are at least secondary school graduates). Attitudes of the husbands and the religious beliefs are not barriers for women's health practices in western Turkey [23]. Since other regions, especially the eastern part of Turkey are more traditional than the western region, migration may be an important marker for attitudes. Health insurance coverage includes routine annual CBE and recommended mammography. Extended families are an indication of traditional life style, and is reflected in siblings, parents and relatives living under one roof.

## Results

According to the women's responses 23.4% of them had no knowledge about breast cancer, 27.9% had no concept of BSE, 89.3% had never had a mammography and 75.0% had never had CBE. 27.9% of the women participants, expressed no previous knowledge of mammography. Only 5.1% of them, had had an annual mammography or over a two year period.

### Knowledge, practice and source of information

Although 72.1% of the participants reported having a knowledge of BSE, only 40.9% of the women in the practised group ever indicated having practised BSE in the previous 12 months. In this BSE practice group, while 29.5% stated they had examined themselves irregularly, only 10.2% stated that they performed BSE on a regular monthly basis. A total of 59.1% of the participants indicated they had never performed BSE. 10.6% of the study group stated that they had had mammography tests and 25.0% had CBE.

A majority of the sample (76.6%) reported that they had heard or read about breast cancer, but only 56.1% of them had sufficient knowledge of it. TV/ radio programs were identified as the main source of information on breast cancer by 39.3% of the participants. Health professionals were mentioned as a source of information by 23.4% of the sample.

### Relationships between sociodemographic variables, beliefs and knowledge of breast cancer

According to logistic regression analysis; the odds of having insufficient knowledge about breast cancer was 2.2 (1.2–4.0) times higher in women who lived in extended families than in nuclear families, 3.7 (1.7–7.9) times higher in women who had never had a CBE than in women had had a CBE and 5.2 (2.5–10.8) times higher in women with no family/friends history of breast cancer than in women with family/friends breast cancer history (Table 2).

Table 3 presents the simple bivariate correlation of each subscale score of CHBMS in relation to the knowledge of the breast cancer score. Significant and positive correlations were found between the knowledge of breast cancer and the perception of confidence in practising BSE (r = 0.58, p < 0.001), BSE-benefit (r = 0.50, p < 0.001), motivation (r = 0.34, p < 0.001) and mammography-benefit (r = 0.50, p < 0.001). Significant negative correlations were also found between the perception of barriers in BSE practice, mammography and knowledge of breast cancer (r = -0.40, p < 0.001) (r = -0.19, p = 0.004) respectively. There were no significant correlations between susceptibility, seriousness domains and levels of knowledge about breast cancer.

### Relationships between sociodemographic and other variables, BSE practice and mammography practice

Table 4 and Table 5 present the relationships between sociodemographic variables, levels of knowledge of breast cancer, BSE practice and mammography practice. A Chi square test was used to evaluate the relationships between BSE and mammography practice with age, education, marital status, health insurance, family type, breast cancer history of family/friends and level of knowledge of breast cancer. The level of knowledge about breast cancer was the only variable significantly associated with the BSE and mammography practice (p = 0.011, p = 0.007).

Table 6 presents comparisons of subscale mean ranks of CHBM on BSE and mammography practice and non-practice groups. A statistically significant finding was seen in the BSE practice group's mean ranks of BSE-benefit and confidence which were higher than in the non-practice group (p = 0.003, p < 0.001). The mean rank of BSE-barrier in BSE-practice was lower than in the non-practice group and this was also found to be statistically significant (p < 0.001). A statistical significance was found between the mean ranks of mammography-benefit, motivation and confidence in the mammography practice group which were seen to be higher than in the non-practice group (p = 0.002, p < 0.001, p = 0.001). The mean rank of mammography-barrier in the mammography practice group was lower than in the non practice group and this was statistically significant (p = 0.033). Susceptibility and seriousness were not significant variables in BSE and mammography, and motivation was not a significant factor in BSE practice.

## Discussion

The literature supports the argument that regular practice of BSE influences treatment, prognosis and survival rates [21,22]. In this study only 10.2% of the participants reported practicing BSE on a regular monthly basis while 29.5% stated that they examined themselves irregularly. Similarly, some studies have reported that less than half of their study groups actually practice BSE monthly [5,24]. In contrast, some studies have found that the majority of older women performed breast screening activities on a regular basis [25,26]. In this study, there was no statistically significant association between the different age groups and BSE. 27.9% of women, expressed that they had no knowledge of mammography and only 5.1% of them, had had an annual mammography over a two year period. The rate of undergoing a recommended mammography practice was lower than Secginli's study which had been performed in Istanbul, and other studies from different countries [4,13,14,28,28].

In this study it was found that 3.3% of women have CBE once a year, and 18.4% when they have a complaint. In the study of Ho, the annual CBE percentage is 45% in educated women [29]. In this study it must be reiterated that 20.1% of our study group were illiterate women living in a rural area.

Although 76.6% of the women in this study reported having heard or read about breast cancer, our study revealed that only 56.1% of them had sufficient knowledge of breast cancer, half of whom had acquired the information from health professionals. Nearly 40% of the study group reported their main source of information on breast cancer was obtained from the TV/radio. This finding indicates the advocacy of TV/radio. Our study group consisted of under educated housewives, to whom TV/radio is readily available which makes it an important information source. It is a considerable finding that health professionals are a relatively poor information source accounting for only 23.4% of the group. Primary health care systems in Turkey is population based. Each midwife/nurse working in primary health care is responsible for a certain groups. Their main responsibilities include child care, pregnancy follow-up and reproductive health problems of women aged 15–49. In this health care system, subjects may be informed about breast cancer screening which in turn may be a solution for women living in a rural areas. According to logistic regression analysis; the odds of having insufficient knowledge about breast cancer was 2.2 (1.2–4.0) times higher in women who lived in extended families than in nuclear families, 3.7 (1.7–7.9) times higher in women who had never had a CBE than in women had had a CBE. A better understanding of the fore mentioned is made possible when taking in to account the knowledge that a majority of the women who live in extended families are more likely to be illiterate and to have a more traditional lifestyle. Champion and Miller have indicated that sociodemographic variables may influence attitudinal variables and thus indirectly influence behavioural patterns. Experiential and demographic variables have several direct paths to attitudinal variables and potential indirect paths to BSE behaviour [30]. In this study it was found that being knowledgeable of breast cancer is the only significant variable in BSE as well as in the practice of mammography. Similarly, in Lagerlund's study, it was found that, a sufficient levels of knowledge of breast cancer had a positive effective factor on having mammography [16]. Hyun's study also reveals that women who are taught to perform BSE have a better level of knowledge about breast cancer [31]. That is to say, variables like age, education level, health insurance, history of breast cancer in friends and/or relatives and family type are not significant in BSE and mammography practice. Similarly, in Jirojwong's study, it was found that sociodemographic variables were not effective on BSE practice [32,33]. Since, independent variables like education levels, women's job status, income level and type of health insurance are similar among the women of this study, they may not be significant in BSE practice as well as mammography.

As in studies of Black American, Australian, Hispanic American, Jordanian, Chinese, Vietnamese, and Asian Indian women, the results of this study indicate that the revised CHBMS is a useful framework to identify factors that influence BSE for Turkish women [4,5,14,17,18,29,33,34].

In this study seriousness, susceptibility and motivation were not significant in explaining BSE performance on a regular basis, but increased confidence, BSE-benefit and reduced BSE-barriers were significantly associated with it. In other studies with Chinese, Jordanian and U.S. women, seriousness has been continuously reported as a non-significant predictor of BSE [18,35-37]. This supports the assertion that seriousness is not a good predictor for preventive health behaviour because breast cancer may be perceived by all women as a serious event, influencing physical, psychological and social aspects of life itself [31].

Motivation was found to be the significant factor for mammography practice in this study. This finding was parallel to the results of Holm and Eun-Hyun [31,38]. In BSE practice, however, motivation was not found to be a significant factor, the practice of monthly BSE was low. One explanation for low compliance is that women in the sample did not believe they were susceptible to breast cancer. Another explanation could be the lack of education in breast cancer (only 56.1% of women had a sufficient level of knowledge). There was no significant association between susceptibility and BSE-mammography practice. The present results parallel those of Eun-Hyun and Petro-Nustas [18,31].

Benefits were a significant variable predicting BSE and mammography performance. It was supported by the findings of American studies that have reported where women who perceived more benefits from BSE behaviour were more likely to perform BSE [14,19,38,39]. In contrast, in other studies with Asian women such as from Korea, Hong Kong and Jordan, benefits were not significant variables [5,26,40].

Confidence was found to be a significant factor for both BSE and mammography practice in this study. The confidence levels of women who perform BSE on a regular basis, and have recommended mammograms, were much higher than among non-practitioners. Research on the relationship between confidence and breast cancer screening has been reported by several researchers with varying degrees of attention to measurement issues. One research group found a positive relationship between confidence and BSE [41]. In a study among Mexican American women, knowledge and confidence were associated with both BSE and colorectal cancer screening [42]. In a few studies, confidence has been related to mammography behaviour. These studies found that, confidence strongly related to a subjects overall intentions to have a mammogram [43]. Similarly, Sortet, Ashton and Foxall's study showed that, women who reported more confidence in performing BSE were significantly more likely to do so regularly [37,44,45].

## Conclusion

Our results indicate that an increase in BSE practice and recommendations for mammograms may be achieved through enhancement of breast cancer awareness and possibly by reducing barriers. By using the CHBMS constructs for assessment, primary health care providers can gain an understanding of the beliefs that influence women's BSE and mammography practice. That of the 76.6% of the sample that reported ever hearing or reading about breast cancer, the majority (39.3%) mentioned TV/radio as their main source of information underscores the potential effectiveness of the media in modifying health behaviour. Health professionals were mentioned as a source of information by 23.4% of the sample. So, the role of health care workers as an information source in breast cancer should be increased. Further research is recommended using a larger sample size with women in rural and urban areas, including the cost-effectiveness of designing and implementing preventive care.

## Competing interests

The author(s) declare that they have no competing interests.

## Authors' contributions

PED conceived of the study, performed the statistical analysis, and drafted the manuscript. DÖ participated in the design of the study and performed the statistical analysis and helped to draft the manuscript. BÖ participated in the design of the study and helped to draft the manuscript. GH, FA, SÇ, GÇ helped acquisition of data and participated in the design of the study. All the authors read and approved the final manuscript.

## Pre-publication history

The pre-publication history for this paper can be accessed here:



## Supplementary Material

Additional File 1 Questionnaire Click here for file

## Figures and Tables

**Table 1:** Sociodemographic features of the study group

	**mean ± sd**
Age	37.7 ± 13.7
Age of first pregnancy	20.8 ± 2.8
Number of living children	2.8 ± 1.4
**Marital status**	**%**
Single	19.7
Currently married	67.6
Widowed/separated	12.7
**Women's occupation**	
Outside work	9.0
Housewife	91.0
**Education level**	
Illiterate	20.1
Primary	49.2
Secondary/above	30.7
**Family type**	
Nuclear	63.1
Extended	33.6
Separated	3.3
**Health insurance**	
Absent	27.5
Present	72.5
**Migration**	
Present	26.2
Absent	73.8
**Perceived family income**	
Sufficient	61.1
Insufficient	38.9
**n = 244**	**100.0**

**Table 2:** OR for level of insufficient knowledge about breast cancer in the study group by family type, breast cancer in family/friends and CBE practice.

	**n**	**OR (95% CI)**
**Family type**		
Nuclear	153	1 (reference)
Extended	91	2.2 (1.2–4.0)
**Breast cancer in family/friends**		
Present	73	1 (reference)
Absent	171	5.2 (2.5–10.8)
**CBE practice**		
Yes	61	1 (reference)
No	183	3.7 (1.7–7.9)

This table includes only the significant variables as determined by the bivariate analysis.

**Table 3:** The correlations between knowledge about breast cancer and subscale scores of CHBMS (n = 244)

**Scale**	**Knowledge on breast cancer**	
	
	**r**	**p**
Susceptibility	0.10	0.1
Seriousness	0.06	0.3
BSE-benefit	0.50	<0.001
BSE-barrier	- 0.40	<0.001
Confidence	0.58	<0.001
Motivation	0.34	<0.001
Mammography-benefit	0.35	<0.001
Mammography-barrier	- 0.19	0.004

**Table 4:** The relations between sociodemographic variables and BSE practice on monthly basis.

**BSE practice on monthly basis**				
**Sociodemographic variables**	**n**	**Present(%)**	**Absent(%)**	**p**

**Education**				
Illiterate	49	4.1	95.9	0.1
Primary/above	195	11.8	88.2	
**Age group**				
15–24	47	2.1	97.9	
25–34	58	13.8	86.2	0.2
35–44	63	9.5	90.5	
45/above	76	13.2	86.8	
**Marital status**				
Married	196	11.2	88.8	0.3
Single	48	6.3	93.8	
**Health insurance**				
Present	197	12.4	87.6	0.1
Absent	4.7	4.5	95.5	
**Family type**				
Nuclear	153	11.1	88.9	0.6
Extended	91	8.8	91.2	
**Breast cancer in family/friends**				
Present	73	12.3	87.7	0.5
Absent	171	9.4	90.6	
**Knowledge on breast cancer**				
Sufficient	137	14.6	85.4	0.011
Insufficient	107	4.7	95.3	

Chi square test

**Table 5:** The relations between sociodemographic variables and mammography practice

	**Mammography practice**			
	**n**	**Present(%)**	**Absent(%)**	**p**

**Education**				
Illiterate	49	5.3	94.7	1.0
Primary/above	195	5.3	94.7	
**Age group**				
15–24	47	0.0	100.0	
25–34	58	1.7	98.3	0.1
35–44	63	7.9	92.1	
45/above	76	9.2	90.8	
**Marital status**				
Married	196	6.6	93.4	0.1
Single	48	48	100.0	
**Health insurance**				
Present	197	6.8	93.2	0.1
Absent	47	1.5	98.5	
**Family type**				
Nuclear	153	4.6	95.4	0.5
Extended	91	6.6	93.4	
**Breast cancer in family/friends**				
Present	73	6.8	93.2	0.5
Absent	171	4.7	95.3	
**Knowledge on breast cancer**				
Sufficient	137	8.8	91.2	0.007
Insufficient	107	0.9	99.1	

Chi square test

**Table 6:** Differences between mean ranks of CHBMS subscale on BSE practice on monthly basis and mammography practice.

	**BSE**			**Mammography**		
				
**Scale**	**practice **(n = 25) mean rank	**non-practice **(n = 219) mean rank	**p**	**practice **(n = 13) mean rank	**non-practice **(n = 231) mean rank	**p**
Susceptibility	133.58	121.24	0.4	118.00	122.75	0.8
Seriousness	122.02	122.55	0.9	87.73	124.46	0.1
BSE-benefit	162.26	117.96	0.003			
BSE-barrier	73.34	128.11	<0.001			
Confidence	188.24	115.00	<0.001	183.54	119.06	0.001
Motivation	133.48	121.25	0.4	191.12	118.64	<0.001
Mammography-benefit				180.85	119.22	0.002
Mammography-barrier				82.31	124.76	0.033

Mann-Whitney U Test

## References

[B1] The Most Frequent Ten Cancers in Females in Turkey, 1999 (online breast cancer resources center). 2005. http://www.saglik.gov.tr/sb/extras/istatistik/apk2001/092.htm.

[B2] American Cancer Society. Cancer Facts and Figures 2005. http://www.cancer.org.

[B3] Lee EO, Ahn SH, You DS, Han W, Choe KJ, Noh DY (2004). Determining the main risk factors and high-risk groups of breast cancer using a predictive model for breast cancer risk assessment in South Korea. Cancer Nursing.

[B4] Sadler GR, Dhanjal SK, Shah RB, Ko C, Anghel M, Harshburger (2001). Asian India women: knowledge, attitudes and behaviors toward breast cancer early detection. Public Health Nursing.

[B5] Fung SY (1998). Factors associated with breast self-examination behaviour among Chinese women in Hong Kong. Patient Education and Counselling.

[B6] Tang TS, Solomon LJ, McCracken LM (2000). Cultural barriers to mammography, clinical breast exam, and breast self-exam among Chinese-American women 60 and older. Preventive Medicine.

[B7] Smith RA, Saslow D, Sawyer KA, Costanza ME, Evans WP, Foster RS, Hendrick E, Eyre HJ, Sener S (2003). American cancer society guidelines for breast cancer screening uptade. Cancer J Clin.

[B8] Lee EH (2003). Breast self-examination performance among Korean nurses. Journal for Nurses in Staff Development.

[B9] Yarbrough SS, Braden CJ (2001). Utility of health belief model as a guide for explaining or predicting breast cancer screening behaviours. Journal of Advanced Nursing.

[B10] Secginli S, Nahcivan (2004). Reliabilty and validity of the breast cancer screening belief scale among Turkish women. Cancer Nursing.

[B11] Zincir H (2000). The knowledge, attitudes and behaviour about breast cancer in 40 year old and older women in Malatya province. PhdThesis.

[B12] Wu TY, Yu MY (2003). Reliability and validity of the mammography screening beliefs questionnaire among Chinese American women. Cancer Nursing.

[B13] Skinner CS, Arfken CL, Sykes RK (1998). Knowledge, perceptions, and mammography stage of adoption among older urban women. Am Journal Preventive Medicine.

[B14] Secginli S, Nahcıvan N (2003). Breast cancer screening behaviors among women. Proceedings of the 2nd International & 9th National Nursing Congress: 07–11 September 2003 Antalya/Turkey.

[B15] Health Education Behavior Models and Theories- A Review of the Literature- Part I. http://msucares.com/health/health/appa1.htm.

[B16] Lagerlund M, Hedin A, Sparen P, Thurfjell E, Lambe M (2000). Attitudes, beliefs, and knowledge as predictors of nonattendance in a Swedish population-based mammography screening program. Preventive Medicine.

[B17] Champion VL, Scott CR (1997). Reliability and validity of breast cancer screening Belie Scales in African American women. Nursing Research.

[B18] Petro-Nustas W, Mikhail IB (2002). Factors associated with breast self-examination among Jordanian women. Public Health Nursing.

[B19] Champion VL (1993). Instrument refinement for breast cancer screening behaviors. Nursing Research.

[B20] Champion VL (1995). Development of a benefits and barriers scale for mammography utilization. Cancer Nursing.

[B21] Nystrom L (2000). How effective is screening for breast cancer. British Medical Journal.

[B22] Facione NC, Giancarlo C, Chan L (2000). Perceived risk and help seeking behavior for breast cancer. Cancer Nursing.

[B23] Hacettepe University, Institute of Population Studies (2003). Turkey Demographic and Health Survey 2003. Ankara.

[B24] Budden L (1998). Registered nurses' breast self-examination practice and teaching to female clients. Journal of Community Health Nursing.

[B25] Smiley MR, McMillan SC, Johson S, Ojeda M (2000). Comparison of Florida Hispanic and non-Hispanic Caucasian women in their health beliefs related to breast cancer and health locus of control. Oncology Nurse Forum.

[B26] Petro-Nustas W (2001). Young Jordanian women's health beliefs about mammography. Journal of Community Health Nursing.

[B27] Champion VL, Mennon U, Mc Quillen DH, Scott C (1998). Validity of self-reported mammography in low-income African-American women. Am Journal of Preventive Medicine.

[B28] Klug SJ, Hetzer M, Blettner M (2005). Screeening for breast and cervical cancer in a large German city: participation, motivation and knowledge of risk factors. European Journal of Public Health.

[B29] Ho V, Yamal JM, Atkinson EN, Basen-Engquist K, Luna GT, Follen M (2005). Predictors of breast and cervical cancer screening in Vietnamese women in Harris County, Houston, Texas. Cancer Nursing.

[B30] Champion VL, Miller TK (1992). Variables related to breast self-examination. Psychology of Women Quarterly.

[B31] Eun-Hyun L (2003). Breast examination performance among Korean nurses. Journal For Nurses In Staff Development.

[B32] Jirojwong S, MacLennan R (2003). Health beliefs, perceived self-efficacy, and breast self-examination among Thai migrants in Brisbane. Journal of Advanced Nursing.

[B33] Savage SA, Clarke V (1996). Factors associated with screening mammography and breast self-examination intentions. Health Education Research: Theory and Practice.

[B34] Austin LT, Ahmad F, McNally MJ, Stewart DE (2001). Breast and cervical cancer screening in Hispanic women: a literature review using the health belief model. Women Health Issues.

[B35] Champion VL (1987). The Relationship of breast self-examination to health belief model variables. Research in Nursing and Health.

[B36] Lu ZJ (1995). Variables associated with breast self-examination among Chinese women. Cancer Nursing.

[B37] Sortet J, Banks S (1997). Health beliefs of rural Appalachian women and the practice of breast self-examination. Cancer Nursing.

[B38] Holm C, Frank DI, Curtin J (1999). Health beliefs, health locus of control and women's mammography behaviour. Cancer Nursing.

[B39] Ruthledge D, Davis G (1988). Breast self-examination compliance and the health belief model. Oncology Nursing Forum.

[B40] Lee YV, Lee EH (2001). Predicting factors of breast self-examination among middle-aged women. Journal of Academy of Adult Nursing.

[B41] Friedman LC, Nelson DV, Webb JA, Hoffman LP, Baer PE (1994). Dispositional optimism, self-efficacy, and health beliefs as predictors of breast self-examination. American Journal of Preventive Medicine.

[B42] Carpenter V, Colwell B (1995). Cancer knowledge, self-efficacy, and cancer screeening behaviors among Mexican-American women. Journal of Cancer Education.

[B43] Champion V, Skinner CS, Menon U (2005). Development of a self-efficacy scale for mammography. Research in Nursing & Health.

[B44] Foxal MJ, Barron CR, Houfek J (1998). Ethnic differences in breast self-examination practice and health beliefs. Journal of Advanced Nursing.

[B45] Ashton L, Karnilowıcz W, Fooks D (2001). The incidence and belief structures associated with breast self-examination. Social Behaviour and Personality.

