# Supplementary material for: The knowledge and attitudes of breast self-examination and mammography in a group of women in a rural area in western Turkey
Source: BMC Cancer. 2006 Feb 24;6:43. doi: 10.1186/1471-2407-6-43 (PMC1403793; doi:10.1186/1471-2407-6-43)
Supplement: Additional File 1 — Questionnaire [file 1471-2407-6-43-S1.doc]

**The Knowledge and Attitudes About Breast Cancer of Women in a Rural Area of Western Region in Turkey**

1. Age--
2. Education level
   1. illiterate
   2. literate
   3. primary school
   4. secondary school
   5. high school
   6. university graduate
3. Women’s job
   1. housewife
   2. worker
   3. civil servant
   4. private sector
   5. student
4. Marital status

1)married

2)single

3)widow

4)separated

1. Husband’s job
   1. workless
   2. worker
   3. civil servant
   4. private sector
   5. student
2. Husband’s education status
   1. illiterate
   2. literate
   3. primary school
   4. secondary school
   5. high school

6)university graduate

1. Family type
   1. core
   2. expanded
   3. separated
2. History of migration
   1. yes
   2. no ( skip to 10 th question)
3. If the answer is yes which region?

1) Western Anatolia 2) Central Anatolia 3)Marmara Region 4)Northern Anatolia 5) Eastern Anatolia 6) Southeastern Anatolia 7) Southern Anatolia

1. The longest living place 1) rural 2) semi-urban 3)urban
2. Perceived family income level

1)sufficient

2)insufficient

1. Health insurance

1)absent 2)poverty card 3)state health insurance 4)private health insurance

1. Menarch age –

Questions For Married

1. gravity --
2. number of live birth --
3. number of stillbirth --
4. number of abortus --
5. number of living child --
6. age at first delivery --
7. duration of breastfeeding (month) --
8. history of menopause 1)yes 2)no (if the answer is no skip to 23rd question)
9. age of menopause --
10. current family planning method

1)absent 2) traditional method (coitus interruptus etc..) 3) modern method

1. İnformation about breast cancer? İf ‘yes’ source of information
   1. no information 2) health professionals 3)books/ brochures/ magazins 4) friends-neighborhood 5)TV-radio 6)other
2. Family history of breast cancer ( you, your mother, sister, aunt , grandmother) / your friends

1)no 2) yes , of my family 3) yes, me 4) yes , my friends

Questions About Knowledge Level of Breast Cancer:

1. What is the effect of aging on breast cancer probabililty?

1)increase 2)decrease 3) no effect 9) don’t know

1. What is the effect of nulliparity on breast cancer probability?

1)increase 2)decrease 3) no effect 9) don’t know

28.What is the effect on breast cancer probability if first delivery age is above 30 ?

1)increase 2)decrease 3) no effect 9) don’t know

29. What is the effect on breast cancer probability if menopaue age is above 50?

1)increase 2)decrease 3) no effect 9) don’t know

30. What is the effect on breast cancer probability if menarch age is under 11?

1)increase 2)decrease 3) no effect 9) don’t know

31. What is the probability of counter-lateral cancer formation in breast cancer patients?

1)increases 2)decreases 3) no effect 9) don’t know

32. What is the effect on breast cancer probability if family history is present?

1)increase 2)decrease 3) no effect 9) don’t know

1. What is the effect of obesity on breast cancer probability?

1)increase 2)decrease 3) no effect 9) don’t know

1. What is the effect of oral contraceptives on breast cancer probability?

1)increase 2)decrease 3) no effect 9) don’t know

1. What is the effect of breastfeeding on breast cancer probability?

1)increase 2)decrease 3) no effect 9) don’t know

1. What is the effect of using alcohol on breast cancer probability?

1)increase 2)decrease 3) no effect 9) don’t know

1. What is the effect of smoking on breast cancer probability?

1)increase 2)decrease 3) no effect 9) don’t know

1. What is the effect of radiation exposure on breast cancer probability?

1)increase 2)decrease 3) no effect 9) don’t know

1. What is the effect of having beningn breast disease on breast cancer probability?

1)increase 2)decrease 3) no effect 9) don’t know

1. What is the effect of hormone replacement therapy on breast cancer probability?

1)increase 2)decrease 3) no effect 9) don’t know

You have knowledge about of which of the following symptoms :

1. bloody discharge from nipple 1) yes 0) no
2. asymmetric sagging in breast 1) yes 0)no
3. breast mass 1)yes 0)no
4. enlargement of neighbouring lymph nodes 1)yes 0)no
5. breast skin retraction 1)yes 0)no
6. abnormal arm swelling 1)yes 0)no
7. nipple retraction 1)yes 0)no
8. discoloration of breast 1)yes 0)no
9. abnormal enlargement of breast 1)yes 0)no
10. Do you know breast self examination? If yes frequency of application?
    1. I don’t know
    2. Yes I know , but never applied
    3. I apply whenever it comes my mind
    4. Once in a month
    5. Others……
11. Do you know what mammography is? If yes frequency of application?
    1. I don’t know
    2. Yes I know , but never underwent
    3. Once in a year
    4. Every two years
    5. Others …..
12. Do you have breast examination by health professionals (CBE- clinical breast examination)? If yes, frequency of CBE ?
    1. never
    2. yes, if I have any complaint
    3. once in a year
    4. others….

**Champion’s Revised Health Belief Model Scales**

*Scale Items*

**SUSCEPTIBILITY**

1. It is extremely likely I will get breast cancer in the future.

2. I feel I will get breast cancer in the future.

3. There is a good possibility I will get bresat cancer in the next 10 years.

4. My chances of getting breast cancer are great.

5. I am more likely than the average women to get breast cancer.

**SERIOUSNESS**

1. The thought of breast cancer scares me.

2. When I thınk about breast cancer, my heart beats faster.

3. I am afraıd to think about breast cancer.

4. Problems I would experience with breast cancer would last a long time.

5. Breast cancer would threaten a relationship with my boyfriend, husband or partner.

6. If I had breast cancer my whole life would change.

7. If I developed breast cancer, I would not live longer than 5 years.

**BENEFITS (BSE)**

1. When I do breast self-examination I feel good about myself.

2. When I complete monthly breast self-examination I don’t worry as much about breast cancer.

3. Completing breast self-examination each month will allow me to find lumps early.

4. If I complete breast self-examination monthly during the next year I will decrease my chance of dyıng from breast cancer.

5. If I complete breast self-examination monthly I will decrease my chances of requiring radical or disfiguring surgery if breast cancer occurs.

6. If I complete monthly breast self-examination it will help me to find a lump which might be cancer before it is detected by a doctor or nurse.

**BARRIERS (BSE)**

1. I feel funy doing breast self-examination.

2. Doing breast self-examination during the next year will make me worry about breast cancer.

3. Breast self-examination will be embarrasng to me.

4. Doing breast self-examination will take too much time.

5. Doing breast self-examination will be unpleasant.

6. I don’t have enough privacy to do breast self-examination.

**CONFIDENCE**

1. I know how to perform breast self-examination.

2. I am confident I can perform breast self-examination correctly.

3. If I were to develop breast cancer I would be able to find a lump by performing breast self-examination.

4. I am able to find a breast lump if I practice breast self-examination.

5. I am able to find a breast lump which is the size of a quarter.

6. I am able to find a breast lump which is the size of a dime.

7. I am able to find a breast lump which is the size of a pea.

8. I am sure of the steps to follow for doing breast self-examination.

9. I am able to identify normal and abnormal breast tissue when I do breast self-examination.

10. When looking in the mirror, I can recognize abnormal changes in my breast.

11. I can use the correct part of my fingers when I examine my breasts.

**HEALTH MOTIVATION**

1. I want to discover health problems early.

2. Maintaining good health is extremely important to me.

3. I search new information to improve my health.

4. I feel it is important to carry out activities which will improve my health.

5. I eat well balanced meals.

6. I exercise at least 3 times a week.

7. I have regular health check-ups even I am not sick.

**BENEFITS- MAMMOGRAM**

1. When I get a recommended mammogram, I feel good about myself.

2. When I get a mammogram, I dont worry as much about breast cancer.

3. Having a mammogram or x-ray of the breast will help me find lumps early.

4. Having a mammogram or x-ray of the breast will decrease my chances of dying from breast cancer.

5. Having a mammogram or x-ray of the breast will decrease my chances of requiring radical or disfiguring surgery if breast cancer occurs.

6. Having a mammogram will help me find a lump before it can be felt by myself or a health professional.

BARRIERS-MAMMOGRAM

1. Having a routine mammogram or x-ray of the breast would make me worry about breast cancer.

2. Having a mammogram or x-ray of the breast would be embrassing.

3. Having a mammogram or x-ray of the breast would take too much time.

4. Having a mammogram or x-ray of the breast would be painful.

5. Having a mammogram or x-ray of the breast would cost too much money.
